# Additional VR-based training improves upper extremity functions in subacute stroke patients: a single-blinded pairwise-matched trial

**DOI:** 10.3389/fneur.2025.1711528

**Published:** 2025-12-19

**Authors:** Milos Dordevic, Cynthia Wendt, Nadine Külzow, Sumit Kundu, Caroline Haun, Bernhard Baier, Anna Gorsler, Notger G. Müller

**Affiliations:** 1 Department of Chronic and Degenerative Diseases, Faculty of Health Sciences (FGW), Potsdam University, Potsdam, Germany; 2 Brandenburg Medical School Theodor Fontane, Neuruppin, Germany; 3 Clinic for Neurorehabilitation, Kliniken Beelitz GmbH, Beelitz-Heilstätten, Germany; 4 Edith-Stein Fachklinik, Bad Bergzabern, Germany; 5 University Medical Center of the Johannes Gutenberg University Mainz, Mainz, Germany; 6 Faculty of Health Sciences Brandenburg, Brandenburg Medical School Theodor Fontane, Brandenburg, Germany; 7 Department of Neurology, Ernst-von-Bergmann Clinic, Potsdam, Germany

**Keywords:** stroke, upper extremity, virtual reality, training, rehabilitation, motor training

## Abstract

**Background:**

Stroke is the leading cause of disability worldwide. Upper extremity paresis is the most common functional consequence, affecting more than half of all stroke survivors. Research has shown that an adequate therapy should begin in the sub-acute stage, but also that an enhanced intensity and frequency of therapy can positively affect patients’ recovery curve. Therefore, here we assessed whether an additional VR-based training can be beneficial for recovery of stroke patients, with particular emphasis on upper extremity functions.

**Methods:**

The study was organized as prospective and single-blinded (analysis). Two groups of pairwise-matched subacute stroke patients with arm paresis were recruited at our rehabilitation clinic while controlling for gender, age, sidedness and modified Rankin scale (mRS). Both groups – conventional therapy (CT) and conventional therapy plus virtual reality (CT + VR) – received 30–45 min of conventional therapy on 3 to 4 days/week over 4 weeks; in addition to that, the CT + VR group received 3 times per week a specially designed VR-based training for upper extremity. Data acquisition was performed within 24 h before the baseline and after the training has ended. Main outcomes were patients’ performance on Fugl-Meyer test for upper extremity (FME), Box-and-Block test, hand dynamometry and Functional independence test (FIM).

**Results:**

Twenty-two subjects aged 57–85 were pairwise-matched and assigned to the conventional therapy (CT) group (*n* = 11, 67.82 ± 8.69 years; three females) and the conventional therapy plus virtual reality (CT + VR) group (*n* = 11, 70.45 ± 6.79 years; three females). No difference in gender, age, sidedness, mRS and mini-mental status examination (MMSE) existed between the two groups. The CT + VR group showed significantly better improvements over time on FME (44.3 ± 7.8 to 58.7 ± 11.2 vs. 42.1 ± 6.2 to 49.5 ± 10.9; *p* = 0.009) and FIM (90.1 ± 18.0 to 118.1 ± 6.9 vs. 105.0 ± 12.4 to 110.6 ± 12.6; *p* < 0.001), compared to the CT group. Other tests revealed no significant differences.

**Discussion:**

As hypothesized, an additional immersive VR-based training can be beneficial for stroke patients suffering from upper extremity deficiency. Nevertheless, the principle of specificity could be observed, with only trained functions being associated with improvements on FME and FIM. Future studies with larger sample of participants are required to confirm these findings.

## 1 Introduction

Stroke is worldwide the third leading cause of death and first of disability, significantly affecting patients’ quality of life and independence (1, 2). Up to 70 percent of all stroke survivors suffer from some grade of upper extremity paresis, with about half of those continuing to have some functional deficits concerning their activities of daily living (ADL) even for years following stroke (3, 4).

Considering that upper extremity impairments are prevalent during both the acute and chronic phases, rehabilitation of post-stroke patients must be considered as a dynamic process involving evaluation, identification, and quantification of patient needs (5, 6). Moreover, many studies agree that high-intensity, adaptive, and task-oriented training is crucial for successful rehabilitation and recovery. However, the therapy intensity recommended by clinical guidelines is often difficult to achieve within the constraints of standard rehabilitation programs (7–9). The early subacute phase, particularly the first 3 months after stroke, represents the period of greatest clinical recovery and is therefore considered the optimal time window for intensive rehabilitation (10). This might be due to increased neuroplasticity in initial recovery stages, indicating that the therapy should begin early (8). However, many persons with chronic stroke have limited opportunities to receive sufficient doses of rehabilitation, because of constraints related to therapist resources and high costs (7). Such treatments are typically supervised by physical therapists and take place in clinics, making them costly and inconvenient to obtain (11). Given the primary goal of neurological rehabilitation – to support recovery from post-stroke deficits and facilitate return to pre-morbid function (2) – approaches that enable patients to train independently without requiring substantial additional staff resources would be highly desirable.

To maximize neuroplasticity and recovery, patients benefit from a well-balanced program that combines structured, institution-based therapies with additional opportunities for self-directed practice. Such add-on options can help maintain motivation, reduce idle time, and promote continuous engagement in the rehabilitation process (12). Recent neurorehabilitation studies employed virtual reality (VR) as a safe (13) and complementary therapeutic tool (14). Immersive VR has been shown to be more effective rehabilitation method than non-immersive VR or gaming consoles (14). Immersive VR combines gaming environment, three-dimensional simulations, artificial intelligence methods and sensor-based technology (9). Therefore, it is able to resemble real environments, allowing interactive and enjoyable neurorehabilitation approaches, leading to an increase in patients’ motivation (4, 5). This is particularly true for immersive VR, which provides more natural and intuitive interaction, with realistic perception of space and level of own presence (11). Its effectiveness in safely improving upper limb motor function and independence in daily life have already been demonstrated (11). In addition, hand-tracking technology – equipped with infrared detectors – was used to provide realistic visual feedback of own hand and finger movements to the patient (6). Our own previous work, using one of the current paradigms, demonstrated comparable neurophysiological responses for immersive VR and observation of own hand movement while the paradigm was played on a computer screen (15). Nevertheless, evidence on potential of this technology in neurorehabilitation of subacute (within 3 months following stroke) patients suffering from various degrees of upper extremity hemiparesis remains very scarce (16). In addition, there are no studies explaining the contribution of an immersive VR-based therapy applied in addition to conventional therapy in this group of patients.

Therefore, we developed specially designed VR-based paradigms for patients with arm paresis, with the intention to administer them an effective add-on therapy in the early subacute phase. All three paradigms were reinforcing hand and arm movements throughout their implementation, in order to accelerate the recovery. The primary goal of this study was to assess the contribution of this additional immersive VR-based treatment on patients’ recovery process, with regards to upper extremity functions and activities of daily life. Hence, we hypothesized that patients who underwent additional immersive VR-based treatment would achieve better recovery outcomes compared to those who received conventional treatment only.

## 2 Materials and methods

### 2.1 Study design and participants

**Figure 1:**
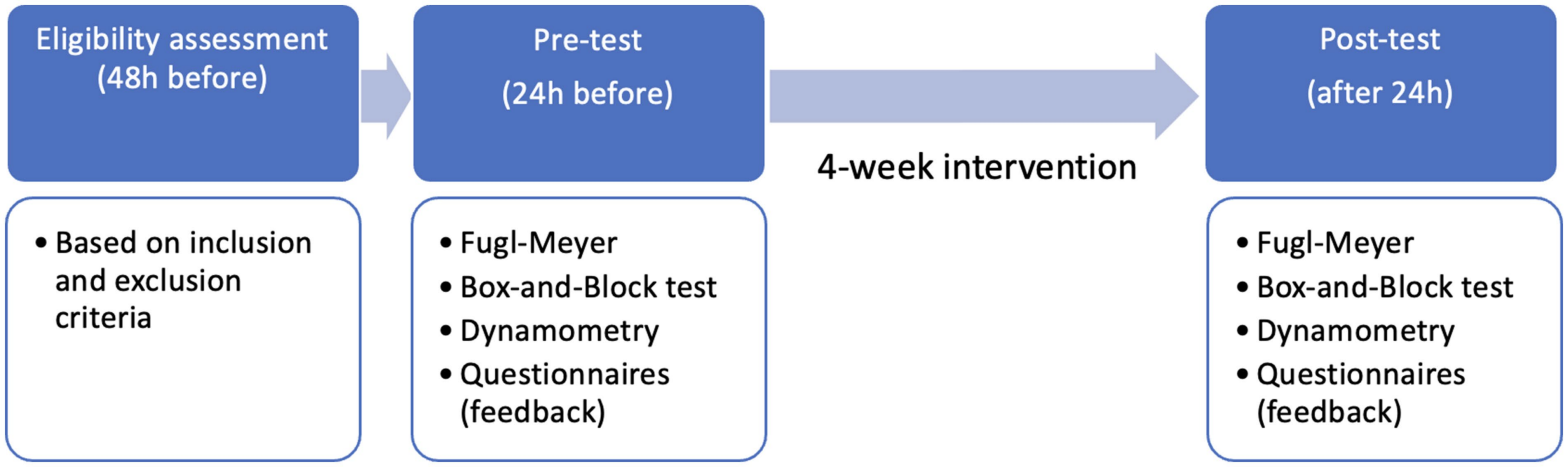
Study design.

This study was organized as a longitudinal pairwise-matched trial, with two groups: (1) conventional therapy (CT) and (2) conventional therapy plus virtual reality (CT + VR) – as depicted in Figure 1. While both groups received the same conventional therapy routine, the CT + VR group received an additional training in virtual reality on 3 days (app. 20 min per day) each week over a period of 4 weeks. Below is a more detailed description of the immersive VR-based setup and paradigms.

**Figure 2:**
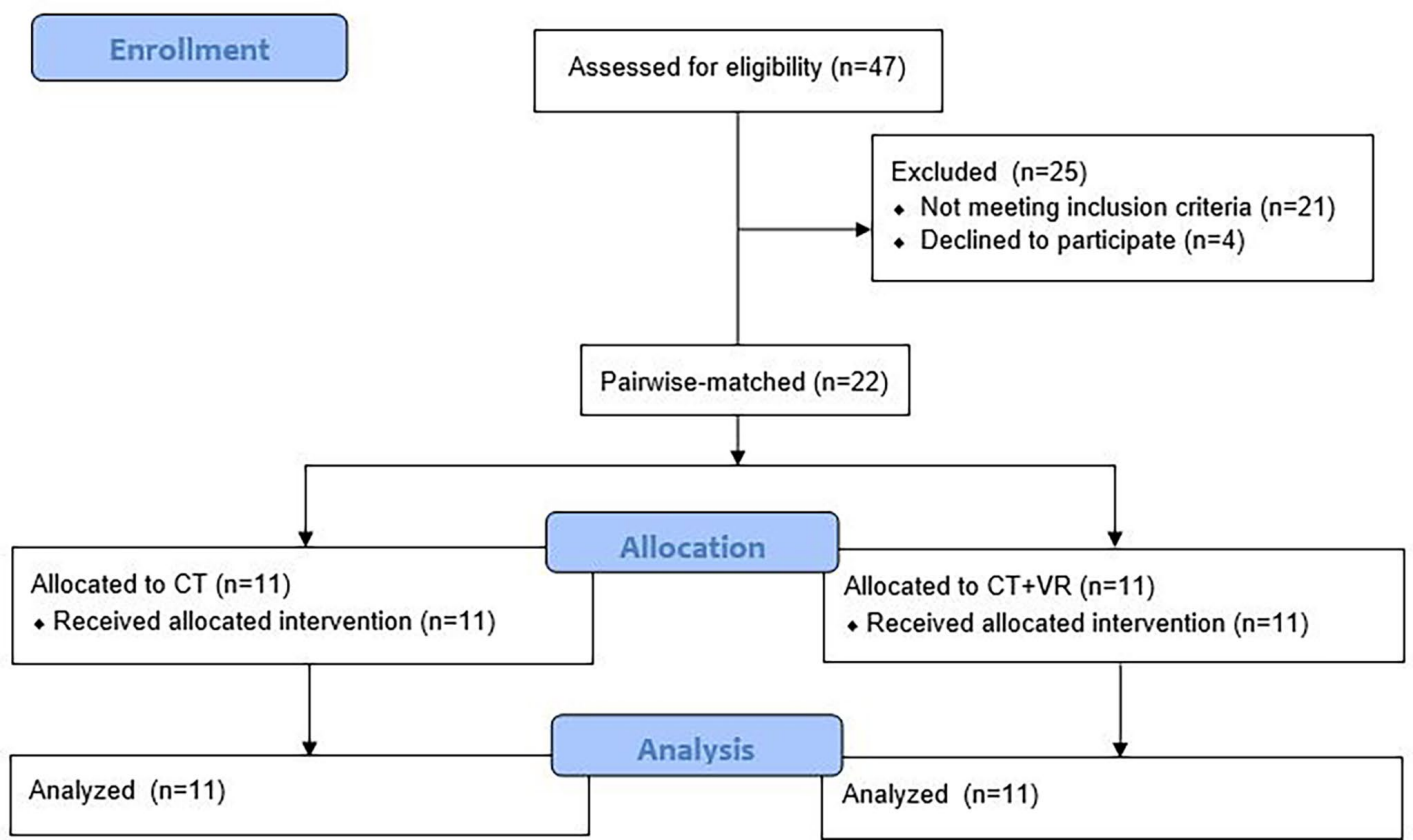
Study flow-chart.

The study was designed in accordance with the declaration of Helsinki and approved by the (approval number 34/2023). After providing written consent, 22 stroke patients (Table 1) were assigned in a pairwise matching manner to the two groups. The pairwise-matching procedure consisted of matching each patient from the CT + VR group to a corresponding patient in the CT group, while controlling for gender, mRS-level, sidedness and age (±3y). The study flow-chart is depicted in Figure 2. Therapists were blinded to patients’ group assignment.

**Table 1:** Characteristics of patients.

Patient	Group	Age	Gender	Side	Level mRS	MMSE
1	CT + VR	80	F	R	3	23
2	CT + VR	80	M	L	3,5	26
3	CT + VR	65	M	L	3	30
4	CT + VR	76	M	L	3,5	29
5	CT + VR	72	M	L	4	24
6	CT + VR	64	F	R	4	26
7	CT + VR	70	M	L	4	30
8	CT + VR	76	M	R	2	27
9	CT + VR	67	F	R	4	28
10	CT + VR	64	M	L	4	28
11	CT + VR	61	M	L	3	27
12	CT	75	F	L	3,5	28
13	CT	85	M	R	4	27
14	CT	57	M	R	3	30
15	CT	63	M	L	3	29
16	CT	76	M	R	4	27
17	CT	69	F	L	4	29
18	CT	70	M	L	4	28
19	CT	61	M	L	2	28
20	CT	71	F	L	4	27
21	CT	59	M	R	4	29
22	CT	60	M	R	3	28

All patients were prospectively recruited for this study through referral from the Clinic for Neurorehabilitation of Kliniken Beelitz GmbH, Brandenburg. Eligible patients were all those who suffered a first unilateral hemispheric stroke involving upper extremity paresis in the last 14 days (early subacute phase), aged from 18 years, were right-handed, were able to understand verbal instructions and give informed consent, were able to sit on a chair for 30 min, belonged to mRS stages 2–4, had no complaints about upper extremity pain and swelling, were medically stable and expected to survive minimum 1 year and achieved MMSE score of 23 or more. Patients were excluded if they had hemispatial neglect, other serious neurological impairment than subacute stroke affecting their ability to perform the required tasks, met DSM-IV criteria for alcohol and substance abuse as well as schizophrenia or other psychotic disorders, serious respiratory or cardiovascular complications, cancer and severe aphasia.

### 2.2 Immersive VR setup and paradigms

**Figure 3:**
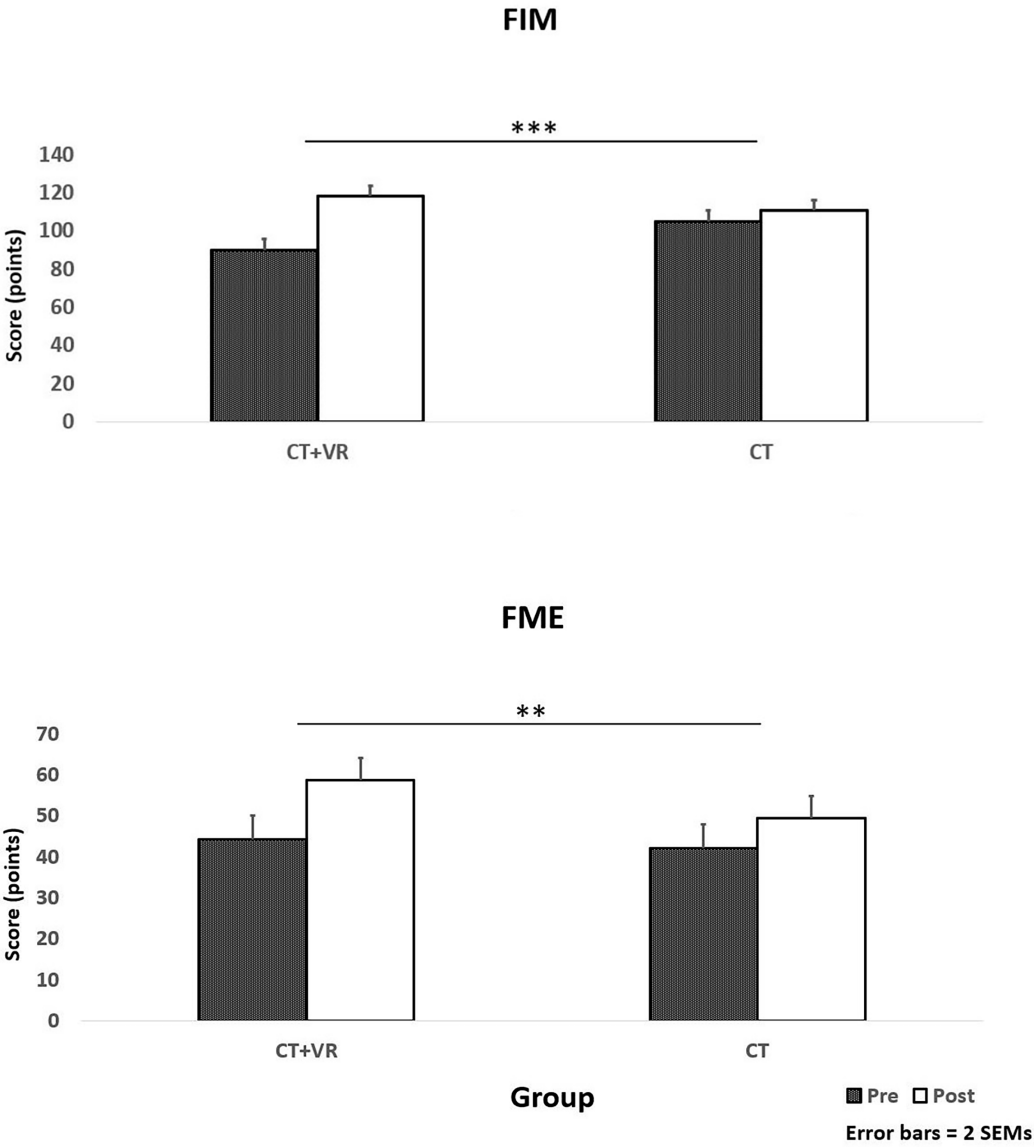
Scores on functional independence measure (FIM, upper panel) and Fugl-Meyer evaluation (FME, lower panel) tests for both study groups and both time-points (pre-post intervention); **p < 0.01; ***p < 0.001.

All measurements took place in the Clinic for Neurorehabilitation of Kliniken Beelitz GmbH (Brandenburg, Germany) from July 2023 until December 2024. A special room was assigned for this purpose, with windows and doors kept closed and blinded for preventing any sound and light interference. After entering the testing room, the whole procedure was explained to the patient, followed by one familiarization trial with each paradigm. Next, the participant was asked to sit in the chair in front of a table and instructed to put the Head-Mounted Display (HMD; HTC-Vive Pro, Taoyuan City, Taiwan) on their head while ensuring optimal adjustments for utmost comfort. On the front side of the HMD, the Leap Motion (Leap Motion Company, San Francisco, CA, United States) controller was placed so that hand movements could be captured within the virtual environment. Once the entire setup was ready, the paradigms were supervised by trained medical student one after the other, in the same order for each participant with each paradigm having the same duration (6 min per paradigm, with 1 min break between). They were informed verbally when to begin and stop performing the task. Three self-developed VR-paradigms were used in this study: (1) bus, (2) football and (3) basket (Figure 3). Each of the three paradigms was created to stimulate and assess motor control using hand gestures in a gamified context, with increasing levels of difficulty to challenge participants over time. Thus, the session progression and adaptive difficulty strategy were considered throughout the study, to match patients’ abilities and maximize motivation. Fine motoric movements, such as those of fingers, were not part of any of the paradigms, because the current state of technology does not allow for continuous and realistic representation of fingers – that is, it may occur that fingers get dipped into a ball during ball grabbing and throwing movements – which will most likely be improved in the near future. For this reason, our paradigms were focusing on arm and hand movements, particularly in shoulder, elbow and wrist joints.

#### 2.2.1 VR-paradigm – bus

**Figure 4:**
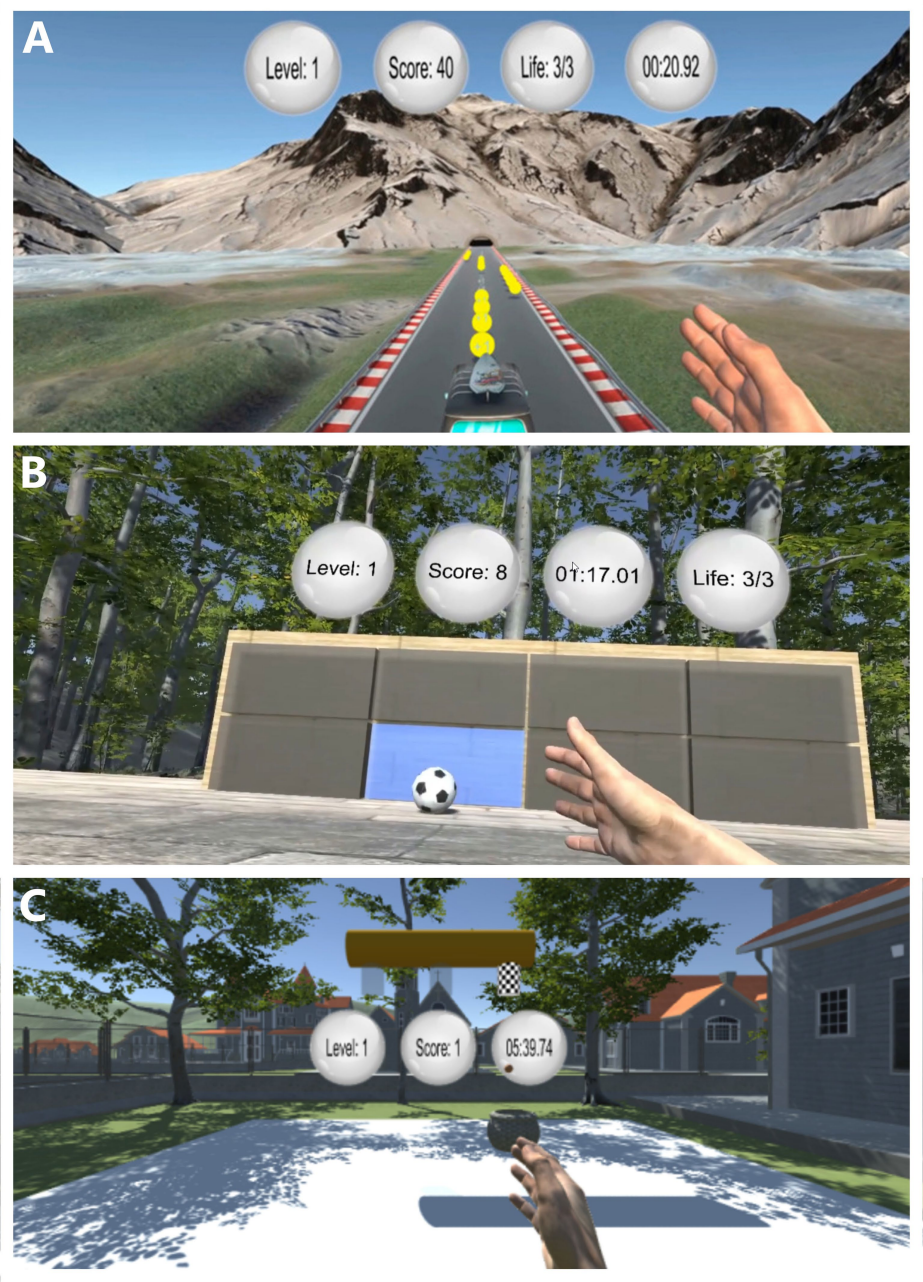
VR-based paradigms. **(A)** bus, **(B)** Football, **(C)** Basket.

This paradigm was specially developed to stimulate arm and hand movements and enhance coordination. As shown in Figure 4A, participants used their hand to steer a moving bus. By holding the hand in sagittal plane and rotating right and left about vertical (wrist) axis, the bus responsively adjusted its position while transitioning between lanes. The participant’s hand remained perpendicular to the lane and hand movements are in palmar- and dorsi-flexion (mainly in the wrist). In addition, participants had to avoid obstacles and collect coins placed on the road. The more the game progressed, the higher the driving speed and thus the difficulty level was achieved. Steering the bus completely off the road lead to re-initiation of the game and return to the starting position. During each run, the goal was to collect a maximum number of points.

#### 2.2.2 VR paradigm – football

In this custom-designed VR paradigm the task for participants was to kick a football toward a target board using hand gestures, as illustrated in Figure 4B. By moving their left or right hand—primarily through wrist flicks involving palmar- and dorsi-flexion, along with wrist and forearm rotations—they propel the ball toward illuminated targets. The hand remains perpendicular to the floor during the action to ensure consistency in movement. Potential targets are presented on a 3 × 3 grid of rectangular fields, with only one of the cells in this grid highlighted in blue (the target), with all other remaining cells being gray (non-target) (see Figure 4B). At any given moment, one of these cells s illuminated to indicate the active target. Participants earn points by accurately hitting the illuminated field using a quick wrist flick or poke gesture. This paradigm also introduces progressive levels of difficulty: (a) Level 1 – only the bottom row of the target grid is used, (b) Level 2, targets are selected from the bottom two rows, and (c) Level 3 fields from any of the three rows may be selected. As the game advance further, additional challenge elements re introduced, such as point deductions for hitting non-target cells and penalties for five consecutive misses. The goal was to maximize the score by accurately and consistently hitting the illuminated targets.

#### 2.2.3 VR paradigm – basket

In this last task, participants had to control a basket using hand movements, as illustrated in Figure 4C. By moving their left or right hand—using the wrist or fingers at the metacarpophalangeal joints—they steer the basket laterally under three designated spawn cylinders. The hand remains perpendicular to the floor, and movements occur through palmar- and dorsi-flexion. The objective is to catch basketballs that fall from the spawn cylinders towards the ground before they touch the ground. As the game progressed, the balls began to fall at a faster rate, and the time intervals between consecutive drops decreased, increasing the task’s difficulty. The goal is to maximize points by successfully catching as many falling balls as possible.

### 2.3 Assessments

All assessments were performed by trained medical personnel within 48 h before the start and after the end of treatment. Assessments took place in the same testing room where patients from the CT + VR group subsequently performed the VR-based treatment. Assessments included following tests: (1) Hand dynamometry, (2) Box and Block (17), (3) Fugl-Meyer Assessment of upper extremity (FME) (18) and (4) Functional independence measure (FIM) (19). Hand dynamometry was performed using the hydraulic hand dynamometer Model HS5001 (SAEHAN Corporation, Republic of Korea) – patients arm was flexed to 90 degrees in the elbow joint (upper arm being vertical, lower arm positioned on chair arm-rest) and the test was repeated three times, with the average value being considered. The box and block test was used to assess hand dexterity of patients – the goal in this test was to move as many cubes as possible within 1 min from one compartment to the other. The upper extremity subset of the Fugl-Meyer evaluation was used in this study, for the purpose of assessing the motor function recovery in the upper extremity following both interventions – in total it consisted of 33 items and a maximal score was 66 points. Finally, the FIM test was used to assess patients’ limitations via performance observation in terms of how much help they needed to perform basic daily activities – this test contains 18 items with scores from 1 to 7 that measure independent performance in physical, cognitive and self-care functions. Final assessment also included an additional questionnaire, in order to obtain feedback from patients on how realistic and user-friendly they found each of the paradigms – on a numeric scale from 1 to 5 (with 5 being optimal).

### 2.4 Data analysis

Data were analyzed using SPSS v.21 (IBM, Armonk, NY, United States) software. Statistical analysis included repeated-measures ANOVAs with time as within-subject and group as between-subject factor and a time × group interaction effect analyses. The significance level was set to *α* = 0.05. The descriptive results are shown as mean ± standard deviation; in addition, effect sizes (*η*^2^*_p_*) and two standard errors of mean of change are reported; the effect size magnitude of ≥0.01 indicated small, ≥0.059 medium and ≥0.138 large effects.

## 3 Results

Complete datasets from 22 stroke patients were obtained and analyzed (Table 1). There were no differences between groups in age (67.82 ± 8.69 years vs. 70.45 ± 6.79 years), modified Rankin-Scale (mRS)-based paresis level (3.50 ± 0.67 vs. 3.45 ± 0.65), mini-mental status examination (MMSE) score (28.18 ± 0.98 vs. 27.09 ± 2.26), gender (both groups consisted of eight males and three females) and side of upper extremity paresis (five right and six left vs. four right and seven left).

Table 2 summarizes the results from all assessments in this study. As shown, a significant group × time effect was found for FME [*F*(1,20) = 8.51] and FIM [*F*(1,20) = 20.49], which assessed corresponding upper extremity functions (namely upper extremity movements) and general independence for self-care, indicating an improvement due to additional VR-based treatment (Figure 4). No significant effects were observed for the other functional outcomes (Box-and-block or hand grip strength).

**Table 2:** Results of all assessments from both study groups (affected extremity only).

Test	Group	Pre-test	Post-test	*p*	*η* ^2^ *_p_*
Dynamometry	CT + VR	19.2 ± 11.1	22.1 ± 12.1	0.438	0.03
	CT	14.2 ± 9.9	15.4 ± 11.3		
Box and Block	CT + VR	33.4 ± 13.7	37.1 ± 15.7	0.945	0.00
	CT	28.5 ± 14.9	32.0 ± 14.1		
FME	CT + VR	44.3 ± 7.8	58.7 ± 11.2	0.009**	0.30
	CT	42.1 ± 6.2	49.5 ± 10.9		
FIM	CT + VR	90.1 ± 18.0	118.1 ± 6.9	<0.001**	0.51
	CT	105.0 ± 12.4	110.6 ± 12.6		

**p < 0.01 (group-time interaction).

Regarding feedback on VR-based paradigms, patients assessed how user-friendly and realistic their experience with these three paradigms was, as follows: (a) Bus – 4.3 ± 0.8 and 4.5 ± 0.5, (b) Football – 4.6 ± 0.7 and 4.5 ± 0.9, and (c) Basket – 4.2 ± 0.9 and 4.4 ± 0.8. Therefore, patients’ feedback ranged from very good to excellent on average, since the scale included values from 0 to 5, with 5 being the best experience.

## 4 Discussion

As hypothesized, this study revealed significantly better recovery of upper extremity (UE) functions and activities of daily living in the group of stroke patients who received immersive VR-based treatment in addition to conventional therapy, compared to those who received conventional therapy only. Moreover, significantly better recovery was detected in relation to motor functions resembling movements in VR-based paradigms, such as hand and arm movements. On the other hand, fine motoric and sensory- or strength-based functions showed no improvement.

Unlike in this study, some earlier studies matched the duration of VR-based therapy with conventional therapy (20). Here we aimed to investigate the contribution of VR-based therapy as an add-on treatment to the already existing conventional routine. Considering that most health- and social-care systems cannot afford to provide an optimal amount of conventional therapy to each stroke patient (21), an add-on VR-based treatment can be seen as one of options. Due to limited resources, patients usually do not receive the conventional therapy intensity and frequency recommended by current guidelines (22, 23). In future, an immersive VR-based add-on therapy could enable patients to increase their training time effectively through self-directed exercises, potentially achieving better functional outcomes. Continuous search for better strategies for recovering upper limb functions following stroke is necessary (24). Khokale et al. already proposed technology-based approaches, including VR, as a way to bridge the gap from provided to optimal amount of therapy for each patient (25). Therefore, a thorough investigation into effectiveness of add-on VR-based therapies is justified. Hence, this study was the first one to show that our specially developed immersive VR-based paradigms can significantly improve the recovery of upper extremity functions in sub-acute stroke patients, when used as an add-on therapy. Better improvement of some relevant upper extremity functions could be seen after only 4 weeks, with three sessions of about 20 min per week, when applied as an add-on to the already existing conventional protocols. This is in accordance with earlier studies, suggesting that VR can be particularly effective in terms of recovery of upper extremity motor function in combination with conventional rehabilitation approaches (26, 27). Also, results of some previous studies do support the use of VR-based paradigms as an augmentation to traditional therapies or as a complementary treatment to conventional therapy (28, 29).

Moreover, many studies consider VR-based treatments as promising tools for recovering upper limb functions and improving daily life activities in stroke patients (24, 30, 31). The majority of studies on immersive VR and upper extremity rehabilitation reported improvements in participants’ rehabilitation outcomes, suggesting that immersive VR may represent a valuable tool for UE rehabilitation in individuals with neurological disorders (32). Our results are in accordance with the findings of a recent review, which reported that immersive VR offers great benefits in rehabilitation, particularly in relation to activities of daily living and functions assessed using the Fugl-Meyer test (33). It is well known that motor functions and manual dexterity rehabilitation benefits, including those achieved through VR-based interventions, are the most pronounced during the acute and subacute stages of recovery, with significant improvements within the first 4 weeks (34–36). Still, based on some other studies, it can be speculated that the observed effects could have been even larger if the patients had been allowed to spend more time in the VR environment (37). Interestingly, it has been shown that customized VR systems can be more effective than commercial ones (38). Although no neurophysiological monitoring assessments were part of this study, it can be speculated that patients’ recovery was based on facilitated neural plasticity and changes in functional connectivity and cortical remapping (39, 40).

Task-specificity has been investigated by numerous previous studies, too. For instance, task-oriented training with an exoskeleton robot has the potential to improve motor functions in a chronically impaired paretic arm even more effectively than some traditional therapies (41). Task-specific rehabilitation protocols are also known to enhance patients’ upper limb performance during the subacute phase of recovery after stroke, both with and without assisting devices (42). In addition, it has been suggested that movement-based priming in combination with task-specific training can lead to even better upper limb recovery, compared to a task-specific training alone (43). When designing an optimal upper extremity rehabilitation protocol, including those VR-based, therapists should try to prevent unnecessary trunk movements and focus on improvement of arm movement quality and function (44). Task-oriented training in hospital may lead to strong improvements in motor functions and performance of daily life activities; in addition, it also leads to moderate improvements when applied in home settings (45). Findings of our current study are also in favor of a task-oriented therapy, since the most significant functional improvements were highly related to those practiced in the VR environment. No floor effects were present in any of the groups, since all patients could actively move their upper extremity.

This study also contains a number of limitations. The main limitation is related to the relatively small sample size; however, this study also represents an initial assessment of our novel immersive VR-based protocol, which is the main reason for working with smaller samples at this stage. Another limitation pertains to the lack of some meaningful neurophysiological information, which could have been obtained via fMRI or fNIRS – insights acquired through these methods would be very valuable when interpreting the results, especially in conjunction with clinical evidence derived from this study. Nevertheless, one review reported that enhanced behavioral changes can be correlated with improved neural plasticity (46), especially when intensive, repetitive and engaging training is provided (47). Still, scientific evidence on VR-based methods should be thoroughly evaluated before such treatments gain widespread use in rehabilitation procedures (48). This should be particularly assessed over longer periods of time. In addition, the examiner in this study was not blinded to patient’s group – considering the subjective nature of tests such as FIM and FME, this could have influenced the recording of scores; however, the medical student was instructed to objectively evaluate each patient and the blinding was performed during the data analysis procedure (single-blinded study). Moreover, no kinematic motor performance tests were included in this study for the purpose of supporting the findings of FME and FIM, which should be considered by future studies. Possible confounders in this study, such as concomitant medications, could have also had an effect patients’ performance – however, all patients were included in the study in both physically and mentally stable conditions. Finally, participants of this study were pairwise matched and not randomly assigned to the two groups, which was not plausible considering the feasibility nature of this study and relatively small sample size – despite that, the applied pairwise matching is a well-known procedure for reliable comparisons between relatively homogeneous groups of patients; however, these findings must be interpreted with caution, considering relatively homogeneous and carefully selected patient groups.

An important observation of this study was that none of the patients experienced any discomfort related to immersive VR-based paradigms, such as sickness or fatigue. This was true regardless of patient’s age, sex, paresis level or any other recorded characteristic. In addition, all patients reported positive subjective feedbacks and interest in further development of this innovative VR-based approach.

In conclusion, this study demonstrated significant and positive effects of immersive VR-based paradigms for functional recovery of subacute stroke patients, suffering from arm and hand paresis. When applied as an add-on treatment to the conventional therapy, better recovery of upper limb functions can be. However, it must be considered that the observed functional improvements highly corresponded with motor functions exercised in VR environment, highlighting task-specificity principle in this type of rehabilitation. Although findings of this study may be beneficial for further clinical projects, larger clinical trials are needed to confirm our findings.

## Data Availability

The datasets presented in this article are not readily available because patient data access is restricted. Requests to access the datasets should be directed to milos.dordevic@uni-potsdam.de.
